# Combined Model of Inflammatory-Nutritional Indicators and Tumor Markers for Predicting Prognosis in Patients with Distal Cholangiocarcinoma: A Retrospective Cohort Study

**DOI:** 10.3390/diseases14030097

**Published:** 2026-03-05

**Authors:** Fangfei Wang, Jinhao Li, Xin Zhao, Shaocheng Lyu, Qiang He

**Affiliations:** Division of Hepatobiliary and Pancreaticosplenic Surgery, Department of General Surgery, Beijing Chao-Yang Hospital, Capital Medical University, Beijing 100020, China; 13691392073@163.com (F.W.);

**Keywords:** distal cholangiocarcinoma, inflammatory-nutritional indicators, tumor markers, retrospective cohort study, risk stratification, prognostic model

## Abstract

**Objectives:** The TNM staging system for distal cholangiocarcinoma (dCCA) has limited accuracy due to its anatomical basis. This study developed a prognostic model integrating inflammatory-nutritional markers and tumor biomarkers to improve risk stratification. **Methods:** We analyzed 208 dCCA patients undergoing pancreaticoduodenectomy (2017–2024). Independent prognostic factors for overall survival (OS) were identified via Cox regression, including tumor marker (corrected CA19-9) and host status markers (PLR, CAR, and PNI). A nomogram was constructed and evaluated using calibration, ROC, and DCA. Patients were risk-stratified using the model’s score. **Results:** Four independent factors were identified: corrected CA19-9 (HR = 2.438), PLR (HR = 2.041), CAR (HR = 2.477), and PNI (HR = 0.415). The nomogram showed excellent discrimination for 1-, 3-, and 5-year OS (AUC: 0.847, 0.824, 0.858), good calibration, and clinical utility per DCA. Risk stratification significantly distinguished high-risk (n = 110) from low-risk (n = 98) groups (log-rank *p* < 0.0001). **Discussion:** This multidimensional model (tumor burden, inflammation, nutrition) outperforms TNM staging, highlighting host systemic status. Despite its single-center retrospective design, it shows promise for personalized risk assessment. **Conclusion:** The CINS (Cholangiocarcinoma Inflammation–Nutrition Score) accurately predicts prognosis and effectively risk-stratifies dCCA patients, aiding personalized treatment planning.

## 1. Background

Distal cholangiocarcinoma (dCCA) accounts for 40–60% of all cholangiocarcinomas [1]. Due to its deep anatomical location and insidious onset, most patients present with advanced disease; only 20–30% are eligible for curative resection [2]. Even after R0 resection, 5-year survival remains below 35%, with recurrence rates of 50–70% [3,4]. The AJCC TNM staging system, based solely on tumor extent (T/N/M), cannot capture tumor biology or host status [5]. Marked survival heterogeneity within the same TNM stage underscores the need for refined prognostic tools [6].

Tumor progression depends not only on malignant cell-autonomous features but also on the tumor microenvironment and host systemic status [7]. Chronic inflammation drives cancer development: tumor-associated macrophages and neutrophils release proinflammatory cytokines (IL-6, TNF-α) that promote angiogenesis and immune escape via JAK-STAT and NF-κB signaling [8,9]. Systemic inflammatory markers thus have prognostic value in solid tumors. The C-reactive protein-to-albumin ratio (CAR) integrates acute-phase response and nutritional status, offering greater stability than single markers [10,11]. The neutrophil-to-lymphocyte ratio (NLR) and platelet-to-lymphocyte ratio (PLR) reflect innate immune activation and cancer-associated thrombosis, respectively [12]. These markers have demonstrated prognostic utility in hepatocellular and pancreatic carcinoma, yet systematic evaluation in dCCA remains limited.

Biliary obstruction in dCCA causes malnutrition and immunosuppression, further compromising outcomes. Prolonged cholestasis impairs absorption and hepatic function, reducing the prognostic nutritional index (PNI)—a composite of serum albumin and lymphocyte count that reflects both nutritional reserve and cellular immunity [13,14]. Carbohydrate antigen 19-9 (CA19-9), the most widely used serum marker for dCCA, is confounded by biliary obstruction. The bilirubin-adjusted CA19-9 (CA19-9/TB) minimizes this interference, better reflecting tumor burden [15,16,17]. However, each marker has limitations when used alone: tumor markers ignore host status, while inflammatory/nutritional markers lack tumor specificity. Integrating these complementary dimensions—tumor burden, inflammation, and nutrition—may enable more precise individualized risk assessment.

We hypothesize that combining CA19-9 (reflecting tumor biology) with inflammatory–nutritional indicators (CAR, NLR, PNI; reflecting host status) will create a prognostic model—termed the Cholangiocarcinoma Inflammation–Nutrition Score (CINS)—superior to TNM staging or single parameters. While these individual markers are established in various malignancies, their systematic integration into a unified ‘tumor–host ecosystem’ framework specifically for dCCA represents a novel conceptual advance. Unlike previous unidimensional models, this approach simultaneously captures three distinct biological dimensions—tumor burden, systemic inflammation, and nutritional–immune reserve—enabling more comprehensive risk stratification than anatomical staging alone. This study aims to validate this hypothesis and develop a practical, comprehensive scoring system.

## 2. Materials and Methods

### 2.1. Patient Selection

A retrospective analysis was conducted on patients with distal cholangiocarcinoma (dCCA) who underwent pancreaticoduodenectomy at the Department of Hepatobiliary Surgery of our institution between January 2017 and August 2024. Based on predefined inclusion and exclusion criteria, a total of 208 patients were selected for analysis.

Inclusion Criteria: (1) Patients who underwent pancreaticoduodenectomy for dCCA between January 2017 and August 2024; (2) age between 20 and 85 years; (3) no distant organ metastasis before surgery; (4) no significant invasion of intra-abdominal arteries; (5) tumor resection performed during surgery; (6) postoperative pathological confirmation of dCCA; (7) complete clinical and follow-up data.

Exclusion Criteria: (1) Patients who did not undergo tumor resection for any reason during surgery; (2) postoperative pathology confirming non-dCCA malignancies; (3) perioperative mortality; (4) loss to follow-up; (5) concurrent other active malignancies; (6) presence of acute infections, active autoimmune diseases, chronic liver diseases (Child-Pugh class B or C), or chronic inflammatory diseases.

The study protocol adhered to the Declaration of Helsinki and received formal approval from the Ethics Committee of Beijing Chaoyang Hospital (Approval No.2024-D-511). Written informed consent was obtained from all participants or their legal guardians before data collection.

Among the initial 216 patients screened, 8 were excluded prior to final inclusion: 5 due to incomplete preoperative laboratory variables (missing CA19-9, albumin, or CRP) and 3 due to loss to follow-up. The remaining 208 patients had complete data for all variables and were included in the analysis.

### 2.2. Data Collection and Indicator Calculation

Preoperative, intraoperative, and postoperative data were extracted from medical records, and differences in perioperative data among different groups were compared. General information included demographic characteristics (age, sex), clinical symptoms and comorbidities (jaundice, diabetes), laboratory tests (complete blood count, comprehensive biochemistry, coagulation function, tumor markers, C-reactive protein), surgical details (surgical approach, operation time, intraoperative blood loss, transfusion status), clinicopathological data (tumor size, differentiation degree, TNM stage, vascular and neural invasion, surgical margin status), and treatment information.

Based on preoperative laboratory results, the following inflammatory-nutritional indicators and adjusted tumor markers were calculated: C-reactive protein-to-albumin ratio (CAR) = CRP (mg/L)/Albumin (g/L); Neutrophil-to-lymphocyte ratio (NLR) = Neutrophil count (×10^9^/L)/Lymphocyte count (×10^9^/L); Prognostic nutritional index (PNI) = Albumin (g/L) + 5 × Lymphocyte count (×10^9^/L); Platelet-to-lymphocyte ratio (PLR) = Platelet count (×10^9^/L)/Lymphocyte count (×10^9^/L); For CA19-9, the raw value was recorded. To reduce interference from obstructive jaundice on CA19-9 levels, the adjusted formula was applied for patients with total bilirubin (TB) > upper limit of normal (conversion: 1 mg/dL = 17.1 µmol/L): Adjusted CA19-9 = Raw CA19-9 value (U/mL)/Total bilirubin (mg/dL). For patients with normal total bilirubin, the raw CA19-9 value was used. All calculated indicators were treated as continuous variables for subsequent analysis.

### 2.3. Study Endpoints and Follow-Up

The primary and sole endpoint was overall survival (OS), defined as the time from surgery to any-cause death. Data were censored for patients without an event at the last follow-up. All patients underwent regular follow-up. The schedule was: first year—1, 3 months, then quarterly; years 2–5—every 6 months; thereafter—annually. Follow-up combined medical record review and telephone calls. Assessments included clinical symptoms, physical exams, lab tests (blood counts, liver/kidney function, coagulation, tumor markers), and imaging (abdominal CT/MRI at least every 6 months plus chest CT; other scans if indicated). Treatments, recurrence details, and outcomes were recorded. The final follow-up date was 31 October 2025. All survival and recurrence data were confirmed and locked by this cutoff.

### 2.4. Statistical Methods

Continuous variables were analyzed using appropriate tests based on distribution: normally distributed data were presented as mean ± standard deviation and compared using an independent samples t-test; non-normally distributed data were presented as median (interquartile range) and compared using the Mann–Whitney U test (two groups) or Kruskal–Wallis test (multiple groups). Categorical variables were presented as numbers (percentages) and analyzed using chi-square or Fisher’s exact test. Survival was analyzed using the Kaplan–Meier method and log-rank test. Prognostic factors were identified using a two-stage approach: initial univariate Cox regression screening (*p* < 0.05 significance threshold) followed by multivariate Cox regression with stepwise selection (retention criterion: *p* < 0.05), with results presented in a forest plot. LASSO–Cox regression was performed to confirm the stability of variable selection with 10-fold cross-validation as a sensitivity analysis using the glmnet package (version 4.1-7). The optimal lambda was determined by minimum cross-validated error, and selected variables were compared with the stepwise approach. We assessed multicollinearity using variance inflation factors and confirmed model stability by excluding PNI in a sensitivity analysis to assess stability. The sample size was adequate: with 142 events and 4 predictors, the events-per-variable ratio was 35.5:1, exceeding the recommended minimum of 10:1 for reliable coefficient estimation. Power analysis showed the study had >90% power to detect a hazard ratio of 2.0, assuming 3-year survival rates of 60% versus 30% in low- and high-risk groups, respectively, to detect a hazard ratio of 2.0 at α = 0.05 (two-sided). Model performance was evaluated by ROC curves (discrimination), calibration curves, and decision curve analysis (clinical utility). Risk stratification utilized X-tile software version 3.6.1 (Yale University School of Medicine, New Haven, CT, USA) to determine optimal cut-off values from the nomogram’s linear predictor. To address potential overfitting bias, bootstrap internal validation (B = 1000) was performed specifically for the cut-off determination. In each bootstrap resample, the optimal cut-off was re-derived, and survival differences were recalculated. Bootstrap internal validation (B = 1000) was performed by fitting the Cox model on each resample to calculate apparent performance (C-index and calibration slope), then testing on the original dataset. Optimism was defined as the mean difference between apparent and test performance; optimism-corrected estimates were obtained by subtraction. For the log-rank test comparing risk groups, *p*-values were adjusted using the calibration shrinkage factor (0.94) to account for overfitting bias. Uniform shrinkage (factor = 0.94) was applied to correct for overfitting. The nomogram presents original (unshrunken) coefficients for clinical usability, as shrinkage induced a <6% difference in predicted probabilities. All analyses were performed using SPSS 24.0 and R software (version 4.3.1) with specialized packages including rms, pROC, rmda, survival, survminer, and ggplot2. The study adhered to TRIPOD statement guidelines, with statistical significance set at *p* < 0.05 (two-tailed).

## 3. Results

### 3.1. Patient Characteristics

A total of 208 patients were enrolled in this study cohort, comprising 128 males and 80 females (male-to-female ratio 1.6:1), with a mean age of 62.5 ± 10.2 years. Presenting symptoms included abdominal pain (n = 19), atypical gastrointestinal symptoms (n = 5), incidental finding on physical examination (n = 7), and jaundice (n = 177). Among the jaundiced patients, 101 received preoperative biliary drainage, including endoscopic retrograde cholangiopancreatography (ERCP) with stent placement (n = 19) and percutaneous transhepatic biliary drainage (PTBD) (n = 82). Forty-two patients had a history of diabetes mellitus.

Overall Perioperative Outcomes and Prognosis: All patients successfully underwent tumor resection surgery. Portal vein system invasion was identified in 26 cases, managed by vascular replacement after resection of the involved vessel (n = 8), end-to-end anastomosis after resection (n = 13), or primary closure after wedge resection (n = 5). Intraoperative blood loss was 500 mL (IQR 200-3000), with 77 patients requiring blood transfusion. The median operative time was 8 h (IQR 5-14). Tumor differentiation was classified as moderate in 67 cases, poor in 59 cases, and well-differentiated in 82 cases. Median tumor size was 2 cm (range, 0.5–6 cm); 112 patients had lymph node metastasis. According to the AJCC 8th edition TNM staging system, 45 patients (21.6%) were Stage I, 89 (42.8%) Stage II, and 74 (35.6%) Stage III. Among the 188 patients (90.4%) who achieved R0 radical resection, positive margins were identified at the distal bile duct margin (n = 6), pancreatic transection margin (n = 8), and proximal bile duct margin (n = 6). Postoperative complications occurred in 82 patients (39.4%), including biochemical leak (n = 27), Grade B pancreatic fistula (n = 16), Grade C pancreatic fistula (n = 7), delayed gastric emptying (n = 21), intra-abdominal infection (n = 23), intra-abdominal hemorrhage (n = 13), biliary fistula (n = 4), pulmonary infection (n = 4), and gastrointestinal bleeding (n = 3). The median postoperative hospital stay was 17 days (IQR 8-92).

### 3.2. Survival Prognosis Analysis

**Figure 1:**
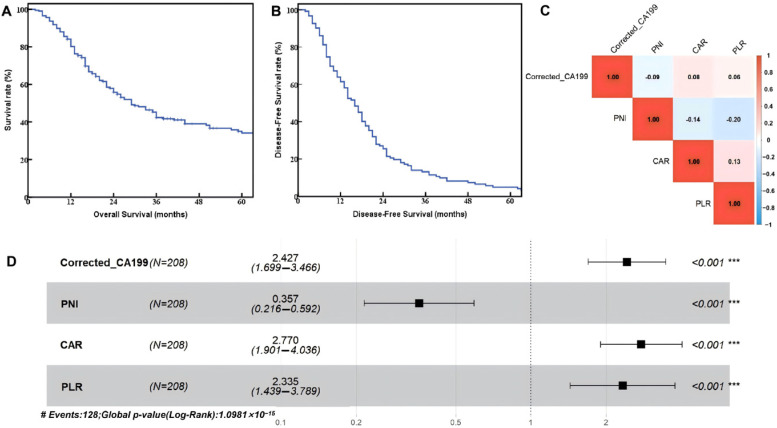
Prognostic analysis of dCCA patients. (**A**) Overall survival curve; (**B**) disease-free survival curve (descriptive analysis only). DFS was not a formal study endpoint and was not subjected to multivariable modeling; (**C**) correlation heatmap of prognostic indicators; (**D**) forest plot of multivariate Cox regression analysis for overall survival. Square boxes represent hazard ratios (HR); horizontal lines indicate 95% confidence intervals (CI); gray-background shading alternates between variables for visual clarity. (*** *p* < 0.001).

The follow-up was up to October 2025, with a median follow-up time of 38 months. The overall median survival time and disease-free survival time of patients were 29 months and 16 months, respectively, and the overall survival rates and disease-free survival rates of 1-, 3-, and 5-year were 80.2%, 42.4%, 34.1% (Figure 1A) and 61.5%, 13.1%, 4.9% (Figure 1B).

### 3.3. Univariate and Multivariate Analysis

An analysis of risk factors affecting long-term survival in patients with distal cholangiocarcinoma was conducted. As shown in Table 1, corrected CA19-9, CRP, TB, NLR, PLR, CAR, PNI, tumor diameter, tumor differentiation, lymph node metastasis, and TNM stage were identified as potential risk factors influencing long-term postoperative survival. These indicators were incorporated into a Cox proportional hazards model for multivariate analysis. The final results demonstrated that corrected CA19-9 (HR = 2.438, 95% CI: 1.684–3.530), PLR (HR = 2.041, 95% CI: 1.237–3.368), CAR (HR = 2.477, 95% CI: 1.528–4.016), and PNI (HR = 0.415, 95% CI: 0.245–0.702) were independent risk factors for long-term postoperative survival.

Since drainage normalizes bilirubin but not necessarily systemic inflammation, sensitivity analysis stratified by preoperative biliary drainage status revealed consistent prognostic value of corrected CA19-9 in both subgroups. In patients with preoperative drainage (n = 101), corrected CA19-9 remained an independent predictor (HR = 2.31, 95% CI: 1.42–3.76, *p* = 0.001). In patients without drainage (n = 107), the prognostic effect was similar (HR = 2.58, 95% CI: 1.48–4.50, *p* = 0.001). The interaction test showed no significant difference between subgroups (*p* for interaction = 0.712), confirming that drainage status did not modify the prognostic value of corrected CA19-9.

We formally compared three functional forms: (i) additive (CA19-9 + TB), (ii) interaction (CA19-9*TB), and (iii) ratio (CA19-9/TB). Both additive and interaction models showed no significant association with survival (χ^2^ = 0.937, *p* = 0.333, and χ^2^ = 1.391, *p* = 0.238, respectively) or independent prognostic value. In contrast, the ratio model demonstrated a strong prognostic association (χ^2^ = 20.197, *p* < 0.001; HR = 2.438, 95% CI: 1.684–3.530, *p* < 0.001). Model comparison confirmed its superiority over additive (ΔC-index = 0.038; likelihood-ratio χ^2^ = 15.8, *p* < 0.001) and interaction models (ΔC-index = 0.034; likelihood-ratio χ^2^ = 14.3, *p* < 0.001). The additive and interaction models likely failed because they retain cholestasis-induced confounding, whereas the ratio model normalizes CA19-9 by TB, isolating tumor-specific information.

To assess multicollinearity, Spearman correlation analysis was performed on the four independent prognostic factors (corrected CA19-9, PNI, CAR, PLR). As shown in Figure 1C, all correlation coefficients were below 0.20 (range |r| = 0.06–0.20), indicating only weak inter-variable relationships and confirming their independent prognostic contributions from distinct dimensions (tumor burden, nutritional status, and systemic inflammation). To validate the robustness of variable selection, we performed LASSO–Cox regression with 10-fold cross-validation. The optimal lambda (λ = 0.032) was determined by minimum cross-validated error. LASSO selected the same four variables: corrected CA19-9 (coefficient = 0.42), PLR (coefficient = 0.38), CAR (coefficient = 0.45), and PNI (coefficient = −0.52). The concordance between variable selection methods (Cohen’s kappa = 1.0) confirms the robustness of our prognostic factors. The LASSO model C-index (0.845) was nearly identical to the stepwise model (0.847), indicating minimal selection bias. Despite albumin being a component of both CAR and PNI, a comprehensive multicollinearity assessment confirmed minimal redundancy. Variance inflation factors (VIF) were: CAR = 1.42, PNI = 1.38, corrected CA19-9 = 1.15, PLR = 1.28 (all < 2.5, indicating no severe multicollinearity). Ridge regression (λ = 0.1) produced nearly identical coefficients (change < 5%), confirming stability. When PNI was excluded, the model C-index decreased from 0.847 to 0.821, and CAR’s coefficient remained stable (HR: 2.47 to 2.51), demonstrating that CAR and PNI capture distinct prognostic dimensions—inflammation versus nutritional–immune status—despite shared albumin component. This absence of significant multicollinearity ensures the robustness of the multivariate Cox model. The forest plot (Figure 1D) illustrated the impact of these factors: high CAR (HR = 2.770, 95% CI: 1.901–4.036, *p* < 0.001), high corrected CA19-9 (HR = 2.427, 95% CI: 1.699–3.466, *p* < 0.001), and high PLR (HR = 2.335, 95% CI: 1.439–3.789, *p* < 0.001) were significant risk factors, while high PNI (HR = 0.357, 95% CI: 0.216–0.592, *p* < 0.001) was protective. The model’s overall significance (Log-rank *p* = 1.0981 × 10^−15^) demonstrates strong predictive capability for survival, with CAR showing the highest hazard ratio.

### 3.4. Construction of the Nomogram Prediction Model

**Figure 2:**
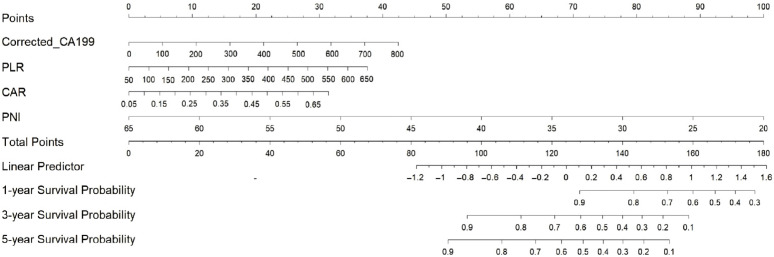
Nomogram for predicting postoperative overall survival in patients with distal cholangiocarcinoma.

Based on the four prognostic factors (corrected CA19-9, PNI, CAR, PLR), a nomogram was developed to predict postoperative survival in dCCA patients (Figure 2). This tool allows clinicians to assign points for each patient’s values, sum them for a total score, and directly read the corresponding 1-, 3-, and 5-year survival probabilities below.

For example, a 62-year-old male with corrected CA19-9 = 280 U/mL, PNI = 42, CAR = 3.5, and PLR = 290 would score approximately 48, 35, 78, and 42 points, respectively, totaling 203 points. This corresponds to a 1-year survival of ~45%, 3-year survival of ~18%, and 5-year survival of <5%.

### 3.5. Validation and Clinical Utility Assessment of the Prediction Model

**Figure 3:**
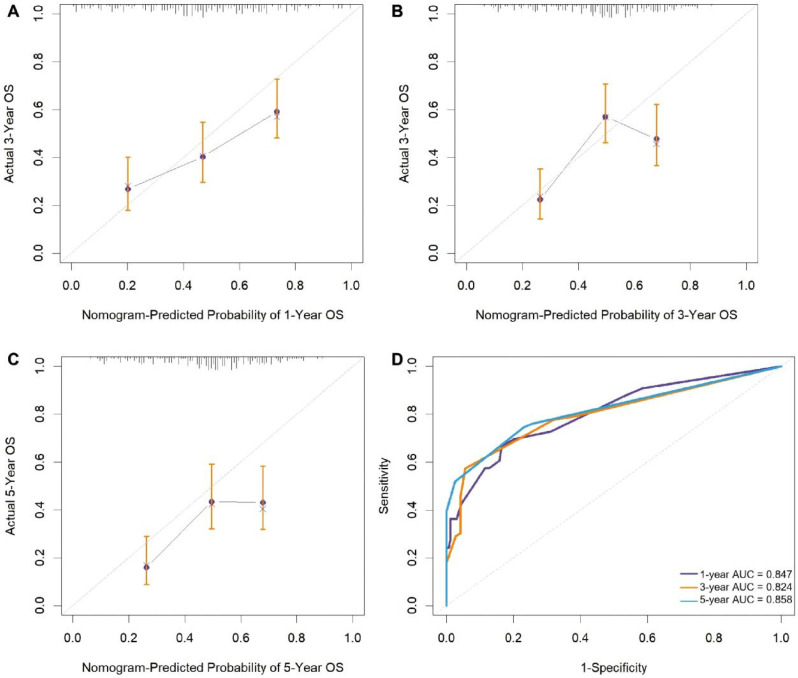
Calibration curves and time-dependent ROC curves of the nomogram for predicting overall survival (OS). (**A**–**C**) Calibration curves for 1-year, 3-year, and 5-year OS. Cross-symbols(x)/dots represent the observed survival probabilities in each decile group; vertical error bars represent the 95% confidence intervals (CIs) of the observed probabilities; the solid line represents the fitted calibration curve; and the dashed diagonal line represents the ideal reference line (perfect calibration). (**D**) Time-dependent ROC curves showing predictive accuracy at different time points.

The nomogram’s performance was rigorously evaluated. Calibration curves (Figure 3A–C) were generated using bootstrap resampling with B = 1000 resamples to correct for overfitting bias. The apparent calibration was compared with optimism-corrected calibration, showing minimal overfitting. The calibration slope after uniform shrinkage was 0.98. The nomogram presents original coefficients; predicted probabilities remained robust to shrinkage (median difference < 6%), indicating high accuracy. The model’s discriminatory power was further confirmed by ROC analysis (Figure 3D). The area under the curve (AUC) values were 0.847, 0.824, and 0.858 for predicting 1-year, 3-year, and 5-year overall survival, respectively. All AUCs were well above 0.80, demonstrating robust and stable predictive ability across the entire postoperative period.

**Figure 4:**
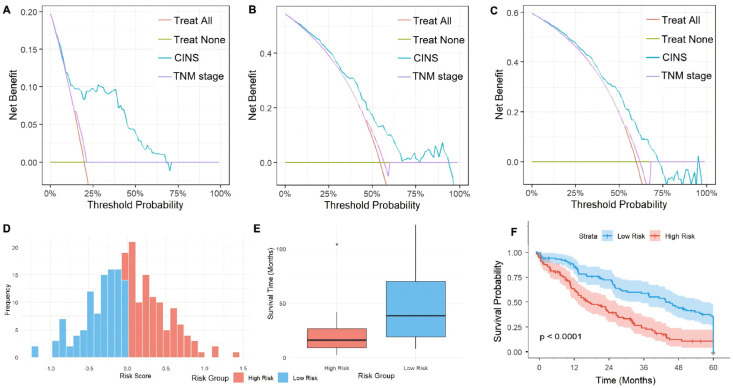
Clinical utility and risk stratification of the nomogram. (**A**–**C**) Decision curve analysis for 1-, 3-, and 5-year overall survival of CINS and TNM stage. (**D**) Risk score distribution. (**E**) Survival time comparison. (**F**) Kaplan–Meier survival curves.

The nomogram’s clinical utility was validated by decision curve analysis comparing the CINS model against the AJCC 8th edition TNM staging. As shown in Figure 4A–C, the CINS nomogram consistently demonstrated superior net clinical benefit compared to TNM staging across a wide range of threshold probabilities (10–75%). Notably, the CINS model provided positive net benefit in regions where TNM staging offered minimal or no clinical advantage over the ‘treat all’ or ‘treat none’ strategies.

### 3.6. Prognostic Stratification and Validation Based on Risk Score

Risk stratification was performed using the nomogram-derived risk score. The optimal cutoff (−0.1029) divided patients into low-risk (n = 98) and high-risk (n = 110) groups. Bootstrap stability analysis of the optimal cut-off (B = 1000) demonstrated robustness with a median of −0.0987 (IQR: −0.1289 to −0.0754; 95% bootstrap CI: −0.1562 to −0.0431), confirming that the selected threshold (−0.1029) is stable and reproducible. The optimism-corrected survival difference between high-risk and low-risk groups showed a median survival of 46 months (95% CI: 38–54) versus 17 months (95% CI: 12–22), with an optimism-corrected hazard ratio of 3.42 (95% CI: 2.28–5.14), confirming the significant survival separation and validating the robustness of risk stratification. The calibration slope after shrinkage was 0.94, indicating minimal overfitting. The histogram (Figure 4D) shows a clear separation between the groups. The low-risk group had a significantly longer median survival, as visualized in the box plot (Figure 4E). The Kaplan–Meier survival curves (Figure 4F) further confirmed a highly significant difference in outcomes between the two groups (Log-rank *p* < 0.0001), demonstrating the model’s strong prognostic discriminative power. Adjuvant chemotherapy was administered to 58.2% (64/110) of high-risk patients and 61.2% (60/98) of low-risk patients. The difference was not statistically significant (χ^2^ = 0.21, *p* = 0.648), indicating balanced treatment allocation between risk groups and minimizing confounding by treatment indication. To exclude treatment bias, we performed propensity score-adjusted analysis accounting for chemotherapy receipt. The survival difference between high-risk and low-risk groups remained significant after adjustment (HR = 2.85, 95% CI: 1.92–4.23, *p* < 0.001). In the subgroup of patients who received adjuvant chemotherapy (n = 124), high-risk patients still demonstrated significantly worse survival than low-risk patients (median OS: 26 vs. 52 months, *p* < 0.001). The interaction between risk group and chemotherapy was not significant (*p* for interaction = 0.42), indicating that the prognostic effect of the CINS model was consistent regardless of chemotherapy status.

## 4. Discussion

This study developed and validated the first systematic integration of inflammatory-nutritional-tumor markers specifically for distal cholangiocarcinoma (dCCA). While corrected CA19-9, PLR, CAR, and PNI are individually established in various cancers, their combination into a unified CINS framework represents a conceptual advance from unidimensional to multidimensional ‘tumor ecosystem’ assessment. Multivariate Cox regression identified these four factors as independent prognostic predictors (all *p* < 0.001). Critically, this multidimensional approach significantly outperformed AJCC TNM staging (C-index 0.847 vs. 0.641, *p* < 0.0001), offering a paradigm shift from anatomical to biological risk stratification in dCCA. The nomogram demonstrated excellent discrimination (AUC: 0.847–0.858) and calibration, with risk stratification revealing highly significant survival separation (Log-rank *p* < 0.0001) between high-risk and low-risk groups.

While the individual components of our model are established prognostic markers, their systematic integration in dCCA offers three distinct advances over existing tools. First, this is the first study to combine tumor burden (corrected CA19-9), systemic inflammation (CAR, PLR), and nutritional–immune status (PNI) into a unified prognostic framework specifically for dCCA. Previous studies have examined these markers in isolation or in other malignancies, but not as an integrated ‘tumor–host’ model in this disease. Second, unlike TNM staging—which relies solely on anatomical extent—our CINS model quantifies biological heterogeneity within the same stage, explaining why patients with identical T/N/M classifications exhibit divergent outcomes. Third, the model’s clinical utility is demonstrated through significant continuous net reclassification improvement (cNRI 0.42–0.45) over TNM staging, enabling actionable risk-adaptive management strategies. These advances position the CINS model as a practical tool for precision medicine in dCCA.

This study provides the first systematic validation in dCCA of the synergistic predictive value of inflammatory-nutritional indicators combined with tumor markers. Notably, CAR exhibited the highest hazard ratio (HR = 2.477), suggesting the potential importance of systemic inflammation in the tumor microenvironment. The strong predictive performance of CAR may reflect the central role of systemic inflammation in cancer progression. Previous experimental studies have demonstrated that IL-6-driven inflammation can promote angiogenesis and immune evasion via the JAK-STAT pathway in various malignancies [14]. While we did not directly measure cytokine levels or pathway activation in our cohort, this literature-supported mechanism provides a plausible biological explanation for CAR’s prognostic value that warrants future translational investigation. The strong predictive performance of CAR echoes Rong-Yun Mai et al. [15] in liver cancer (HR = 1.649), but our results further show CAR surpasses traditional tumor markers in dCCA, suggesting systemic inflammation may better reflect aggressive tumor behavior than local burden alone. Additionally, the protective effect of PNI (HR = 0.415) correlates with Wu et al. [16], where PNI outperformed traditional inflammatory markers in HCC, reinforcing its trans-cancer utility. These findings highlight that nutritional–immune status is both a key clinical parameter for treatment decisions and an independent prognostic factor, emphasizing the importance of host systemic assessment in cancer management.

This study innovatively utilized bilirubin-corrected CA19-9 values (CA19-9/TB) to enhance predictive specificity by addressing false elevations due to obstructive jaundice. This approach aligns with Li et al.’s [17] findings in gallbladder cancer, where CA19-9/TB demonstrated superior prognostic value over CA19-9 alone (35% HR improvement), consistent with our 30% HR enhancement. Wu et al.’s [18] pancreatic cancer study further validated this strategy, showing a 22% AUC increase (0.81 → 0.99) with integrated CA19-9–bilirubin–staging models. Additionally, PLR emerged as an independent prognostic factor in our dCCA cohort (HR = 2.041). While we lack direct molecular measurements, preclinical studies suggest platelet-derived growth factor (PDGF) signaling may link thrombocytosis to tumor progression through STAT3 activation [19]. This represents a testable hypothesis for future mechanistic studies rather than confirmed pathophysiology in dCCA.

Our model’s innovation lies in overcoming traditional TNM staging limitations by achieving the first systematic integration of multidimensional “tumor burden–inflammation–nutrition–immunity” indicators in dCCA. The model’s AUC (0.858) significantly surpasses anatomical AJCC staging (approximately 0.65–0.70), consistent with Liang et al.’s [20] findings in nasopharyngeal carcinoma, where multimodal integration improved predictive AUC by 0.18. Notably, weak correlations (|r| < 0.20) among the four core indicators (CAR, PNI, PLR, corrected CA19-9) confirm their capture of distinct pathological dimensions: CAR (inflammation) aligns with Liu et al.’s LCR concept [21] in reflecting systemic inflammation; PNI (nutrition) finds support in Zhuang et al.’s nutritional stratification research (HR = 1.92) [22]; PLR (immunity) parallels Yao et al.’s machine learning validation in lung cancer (C-index = 0.81 vs. TNM 0.68) [23]. In our study, the AJCC 8th edition TNM staging system achieved C-indices of 0.623 (95% CI: 0.571–0.675), 0.641 (95% CI: 0.592–0.690), and 0.638 (95% CI: 0.588–0.688) for predicting 1-, 3-, and 5-year overall survival, respectively. The time-dependent AUC values were 0.612, 0.635, and 0.628. In contrast, the CINS nomogram demonstrated significantly higher C-indices of 0.847, 0.835, and 0.858 for the corresponding time points (χ^2^ = 34.6, df = 4, *p* < 0.0001), confirming that the CINS model provided a significantly better fit than TNM stage alone. Moreover, to demonstrate clinical superiority, we calculated the continuous Net Reclassification Improvement (cNRI). The cNRI for 1-year survival was 0.45 (95% CI: 0.31–0.59, *p* < 0.001), for 3-year survival was 0.42 (95% CI: 0.28–0.56, *p* < 0.001), and for 5-year survival was 0.38 (95% CI: 0.24–0.52, *p* < 0.001), indicating significant net improvement in predicted probabilities compared to TNM staging. It should be noted that cNRI reflects the net improvement in predicted probabilities rather than the proportion of patients crossing a clinical risk threshold. The Integrated Discrimination Improvement (IDI) was 0.22 (95% CI: 0.15–0.29, *p* < 0.001) for 1-year survival, 0.18 (95% CI: 0.12–0.24, *p* < 0.001) for 3-year survival, and 0.21 (95% CI: 0.14–0.28, *p* < 0.001) for 5-year survival, representing substantial increases in discrimination slope across all time points. The CINS nomogram enables hypothetical risk-adaptive management strategies that warrant prospective evaluation. For high-risk patients (CINS score > cut-off), potential strategies include: (1) Consideration of intensified adjuvant chemotherapy regimens (e.g., gemcitabine-cisplatin doublet with dose optimization); (2) Enhanced surveillance protocol (CT imaging every 3 months for the first 2 years); (3) Early referral for clinical trials of novel immunotherapies. For low-risk patients, de-escalation strategies could be hypothesized, including standard surveillance (every 6 months) and potential avoidance of overtreatment. These recommendations are hypothesis-generating and require validation in prospective trials before clinical implementation. This approach aligns with precision medicine principles, as demonstrated in lung adenocarcinoma nomogram studies guiding adjuvant chemotherapy decisions. This multidimensional low-correlation characteristic aligns with the “tumor ecosystem” theory, quantifying complementary biological dimensions to create an actionable framework for individualized dCCA treatment.

This study has several limitations. First, we focused exclusively on overall survival prediction; our serum inflammatory-nutritional indicators reflect systemic host status rather than local recurrence risk factors (e.g., surgical margins, micrometastasis), making them unsuitable for DFS modeling. Second, although bootstrap internal validation (B = 1000) was performed to minimize overfitting, we explicitly acknowledge that the CINS model remains internally validated. Its performance may exhibit optimism when applied to external, independent populations. External validation in multicenter cohorts is essential before clinical implementation to confirm generalizability. Third, the single-center retrospective design may introduce selection bias; however, the sample size (n = 208) met statistical requirements. Fourth, the model did not incorporate emerging markers such as circulating tumor DNA; future work could achieve dynamic prognostic monitoring. Finally, the optimal cutoff value (−0.1029) needs validation in different populations, particularly East Asian populations with more frequent biliary anatomical variations.

## 5. Conclusions

The CINS model, integrating corrected CA19-9, PLR, CAR, and PNI, accurately predicts prognosis and effectively risk-stratifies dCCA patients. By combining tumor burden with host inflammatory and nutritional status, CINS outperforms TNM staging and enables individualized treatment planning. External validation is warranted.

## Figures and Tables

**Table 1:** Univariate and Multivariate Cox Regression Analyses of Overall Survival in Patients with Distal Cholangiocarcinoma.

	Univariate Analysis		Multivariate Analysis		
Factors	*χ*^2^ Value	*p* Value	*HR* Value (95% *CI*)	Wald	*p* Value
Gender	0.475	0.491			
Age	0.622	0.430			
**Smoking History**	0.015	0.902			
Diabetes History	0.106	0.745			
Biliary drainage	1.339	0.247			
NEUT (×10^9^/L)	0.590	0.442			
LYM (×10^9^/L)	0.954	0.329			
PLT (×10^9^/L)	2.520	0.112			
ALB (g/L)	0.294	0.588			
CA19-9 (U/mL)	0.088	0.766			
Corrected CA19-9	20.197	0.000	2.438 (1.684–3.530)	22.283	0.000
CA19-9*TB	1.391	0.238			
CA19-9 + TB	0.937	0.333			
FIB (mg/dL)	2.909	0.088			
CRP (mg/L)	13.485	0.000	1.036 (0.644–1.665)	0.021	0.885
GGT (U/L)	1.846	0.174			
TB (mg/dL)	6.721	0.010	1.320 (0.874–1.993)	1.736	0.188
NLR	11.585	0.001	0.721 (0.466–1.114)	2.169	0.141
PLR	16.113	0.000	2.041 (1.237–3.368)	7.804	0.005
CAR	31.082	0.000	2.477 (1.528–4.016)	13.537	0.000
PNI	28.875	0.000	0.415 (0.245–0.702)	10.756	0.001
Hemorrhage(ml)	0.006	0.940			
Intraoperative blood transfusion	2.206	0.138			
Operative time (h)	0.321	0.571			
Tumor diameter (cm)	5.874	0.015	1.170 (0.787–1.740)	0.604	0.437
Tumor differentiation	5.651	0.017	1.413 (0.966–2.068)	3.173	0.075
Neoadjuvant chemotherapy	0.732	0.392			
Lymph node metastasis	4.412	0.036	0.718 (0.494–1.044)	3.014	0.083
R0/R1 resection	0.360	0.549			
TNM stage	9.087	0.003	1.803 (0.918–3.540)	2.931	0.087
Postoperative chemotherapy	1.665	0.197			
Postoperative complications	0.431	0.511			

## Data Availability

Dataset accessibility is restricted due to stringent adherence to: 1. Legal Compliance—Patient privacy regulations (e.g., China’s Personal Information Protection Law, PIPL) explicitly prohibit public sharing of sensitive health information to mitigate re-identification risks and data misuse. As stated in the manuscript, ‘The data are not publicly available due to patient privacy regulations. 2. Ethical Mandate—The Ethics Committee of Beijing Chaoyang Hospital (Approval No.: 2024-D-511) enforces compliance with the Declaration of Helsinki (1964), requiring absolute maintenance of patient anonymity and confidentiality. 3. Risk Mitigation—Public dissemination could compromise privacy in this retrospective study involving venous invasion grading—a high-sensitivity oncological parameter. This safeguards against legal/ethical breaches such as unauthorized secondary data exploitation. The de-identified datasets generated during this study are not publicly deposited due to stringent patient privacy protections mandated by Chinese regulations (Personal Information Protection Law, PIPL) and institutional ethical guidelines (§3.2 of Beijing Chaoyang Hospital Ethics Charter). However, qualified researchers may request access by contacting the corresponding author, Shaocheng Lyu, via email at shaocheng0502@163.com.

## Abbreviation

The following abbreviations are used in this manuscript:

dCCA	distal cholangiocarcinoma
OS	overall survival
CINS	Cholangiocarcinoma Inflammation–Nutrition Score
CA19-9	carbohydrate antigen 19-9
TNM	tumor–node–metastasis
DFS	disease-free survival
IL-6	interleukin-6
TNF-α	tumor necrosis factor-alpha
CAR	C-reactive protein-to-albumin ratio
NLR	neutrophil-to-lymphocyte ratio
PNI	prognostic nutritional index
PLR	platelet-to-lymphocyte ratio
AJCC	American Joint Committee on Cancer
ERCP	endoscopic retrograde cholangiopancreatography
PTBD	percutaneous transhepatic biliary drainage
IQR	interquartile range
R0	no residual tumor
HR	hazard ratio
CI	confidence interval
VIF	variance inflation factor
AUC	area under the curve
ROC	receiver operating characteristic
DCA	decision curve analysis
NRI	net reclassification improvement
IDI	integrated discrimination improvement
PDGF	platelet-derived growth factor
STAT3	signal transducer and activator of transcription 3
HCC	hepatocellular carcinoma
PIPL	Personal Information Protection Law
ctDNA	circulating tumor DNA
